# Is the undergraduate microbiology curriculum preparing students for careers in their field?: an assessment of biology majors’ conceptions of growth and control of microorganisms

**DOI:** 10.1186/s40594-018-0138-z

**Published:** 2018-10-19

**Authors:** Aakanksha Purushottam Sawant, Swapnaja Arvind Patil, Jyotsna Vijapurkar, Needa Nasir Bagban, Deepti Bhushan Gupta

**Affiliations:** 1 Homi Bhabha Centre for Science Education (Tata Institute of Fundamental Research), V.N. Purav Marg, Mankhurd, Mumbai, 400088 India; 2 Present Address: Wildlife Conservation Trust, Mafatlal Center, Nariman Point, Mumbai, 400021 India

**Keywords:** Undergraduate biology, Alternative conceptions, Microbiology, Curricular reforms, DBER

## Abstract

**Background:**

We present an analysis of students’ responses to application-based questions on the topic of growth and control of microorganisms, from a questionnaire administered to 348 second and third year students of an Indian university who were enrolled in its undergraduate programs in Biotechnology or Microbiology. We examined aspects of the laboratory practice as reported by teachers and of the university assessment patterns that may explain our findings. Reports by teachers also included their views on the impact of the laboratory curriculum on building student capabilities. Studies such as this play an important role in informing the ongoing discourse in the country about much-needed reforms in undergraduate education.

**Results:**

Our analysis revealed several lacunae in students’ understanding. Students’ performance on the questionnaire was also found to be poorly correlated with their academic achievement in the university examinations. Teachers’ reports revealed that there was a minimal student involvement in planning and designing of the experiments in their laboratory course; rather, cookbook protocols were commonly used by the students. There was a striking disparity between students’ stated career aspirations and their preparedness for them.

**Conclusions:**

Our analysis points to underlying issues in the teaching-learning and assessment process; we discuss these issues and possible alternatives to the current practices. This study, to the best of our knowledge, is the first in the country that has explored students’ conceptions for an elementary topic in biology education at the tertiary level. We believe that the results of the study will be useful in shaping the ongoing educational reforms in higher education and will also be useful in developing a concept inventory on this topic.

**Electronic supplementary material:**

The online version of this article (10.1186/s40594-018-0138-z) contains supplementary material, which is available to authorized users.

## Background

The need for reforms in higher education in India has been widely acknowledged. In the recent past, several reports have summarized the state of higher education in India and emphasized the need for major reforms at both structural and functional levels (Sen 2015; Department of Higher Education (DHE) 2015; University Grants Commission (UGC) 2016; Saidapur 2017; Nityananda 2017). Some reforms have been implemented, and many others proposed, as discussed in Balaram (2010, 2013). While the need for reform has been recognized, there has nonetheless been a dearth of discipline-specific empirical studies reporting on aspects of the current system. One such study by Phadnis and Pandit (2011) evaluated the impact of a conventional assessment system on the teaching-learning process in the country. The study reported that the current assessment routines are pushing students towards “rote learning” and fostering a culture of “surface learning” suitable only for scoring well in the University examinations. It also pointed to deficiencies in the curriculum which offers little scope for learning by doing. There is an urgent need to build a repository of such studies rooted in research, both qualitative and quantitative, to reveal the problems of the existing education system and thus inform the reform process. As can be expected, many science education reforms around the world have focused on helping students achieve conceptual understanding (American Association for the Advancement of Science (AAAS) 2001; National Research Council (NRC) 2012). Studies diagnosing students’ understanding form the first step in the process of such reforms; the nature of ideas presented by students, particularly when they deviate from those that are scientifically accepted, can provide insights into formative steps of knowledge construction by students and serve as feedback for improving the teaching and learning process (diSessa and Sherin 1998; Tanner and Allen 2005). Indeed, a large repertoire of studies identifying misconceptions/alternative conceptions in biology highlights the importance of such studies in determining the origin and structure of these diverse ideas (Fisher 1985; Michael et al. 1999; Nehm and Reilly 2007; Pinarbasi 2007; Cooper and Shore 2008).

We have recently embarked on a project designing interventions in the teaching-learning process that are practicable at the undergraduate level in India. As part of that project, we explored second year undergraduate students’ understanding of some basic concepts in biology—growth and control of microorganisms being one of them. Most experiments in microbiology require cultivation of microorganisms at one stage or another; students need to thoroughly understand the growth kinetics of a microbial culture to be able to quantify the microbial population for basic research and industrial applications. Indeed, the study of bacterial growth has long been considered an essential foundational concept in microbiology, one that all learners are required to master both in theory and practice (Monod 1949; Egli 2015). Of equal importance to microbiology is the control of microorganisms: the process of disinfection and sterilization. In microbiology, sterilization refers to the complete destruction or elimination of all viable organisms in or on a substance being sterilized. There is no degree of sterilization: An object or a substance is either sterile or not. On the other hand, disinfection refers to the partial removal of, or a significant decrease in, the microbial load. Depending on the time of contact, the type, and the concentration of the disinfectant being used, there may be high levels of disinfection but that is not the same as sterilization. It is crucial for a student enrolled for a degree in biology to understand the difference between the two processes to ensure decontamination of laboratory equipment and growth media. They also need to understand the mode of action of each method to determine its suitability for the material to be sterilized or disinfected. These foundational concepts of growth and control of microorganisms are intricately linked and are covered in fair detail in both theory and laboratory curriculum in the first year of both Microbiology and Biotechnology majors in Mumbai University (and in general in universities across the country). The laboratory syllabus for all 3 years has several experiments that require the application of these concepts (University of Mumbai n.d). This syllabus is followed by all colleges affiliated to Mumbai University and, since 2017, all students undertake common assessments, semester wise, conducted by the University.

We report here our analysis of students’ responses to questions on the topic of growth and control of microorganisms. The study was extended to record teachers’ views on the curriculum and their general approach to laboratory sessions. We also report teachers’ opinions of the professions their students were competent to take up after the completion of the 3-year undergraduate degree course. We compare this with students’ own reports of their career aspirations.

The major research question that guided this study was:

What conceptions do undergraduate biology students have regarding the growth and control of microorganisms?

The subsequent extension of the study was conducted to answer these research questions:

What are the curricular issues, if any, that might be impeding the development of conceptual clarity and higher order thinking skills in this topic among students?
(a) Are students’ career aspirations aligned with their conceptual understanding of the topic?
  (b) What are the teachers’ perspectives on the skills and capabilities that the undergraduate course is developing in students?

To the best of our knowledge, this is the first discipline-specific study in the country that has investigated student ideas about this foundational concept. We believe that our study can provide insights for improving teaching-learning for this topic, thus contributing towards undergraduate biology education reforms in the country. The insights into alternative conceptions held by students may also be useful for building tools such as concept inventories. A recently developed concept inventory in microbiology (Paustian et al. 2017), though quite extensive, could be made more robust by including this concept.

## Methods

### Participants in the study and data collection

Four groups of participants, two of students and two of teachers, volunteered to be a part of the study. The first group consisted of 296 students in the second year (SY), and the second group consisted of 52 students in the third year (TY) of their degree course. These students, typically aged 19–21 years, came from five colleges, affiliated to the same university, following the same curriculum, and were enrolled in a 3-year degree program in Biotechnology (BT) or Microbiology (MB). These colleges were selected because they spanned a range of minimum eligibility scores (Table 1) required for admission to the bachelor’s program (B.Sc.) (~ 40–82% aggregate score in grade 12).

**Table 1:** College wise data on the number of participating students and stated minimum eligibility score

College		I	II	III	IV	V (SY + TY)
Minimum eligibility score (%)		42	54	70	82	60
No. of students	MB	42	35	38	37	29 + 32
	BT	13	28	33	26	15 + 20

*SY* second year, *TY* third year

Eligibility to enroll in the undergraduate program is based on the grade 12 scores in examinations conducted by boards of education across the country. Copies of mark sheets for grade 12 and the first year of B.Sc. were obtained from students and the minimum eligibility score for admission from the college authorities. For more details on the distribution of participants’ grade 12 and first year marks (in their discipline—MB or BT), see Additional file 1: Figure S1.

Due to logistical constraints such as college schedules, the study was spread over nearly a year. Since the questionnaire was administered to college V towards the end, the second year B.Sc. students from that college had already graduated to the third year of B.Sc. at the time of administering the questionnaire. So, in addition to the second year students, it was administered to the third year students as well. Our motivation to include them was also that they had undertaken other advanced courses such as Industrial Microbiology/Biotechnology (which discuss the industrial production of cheese, wine, etc.), where sterilization of media/equipment and growth of microorganisms are important base concepts. Third year students’ responses could act as an indicator of improvement in understanding, if any, after having revisited the same concepts.

Two questionnaires were administered to all students (group 1 + group 2)—the first questionnaire checked their level of conceptual clarity and included a section on feedback on the difficulty level and the language of the questions (see Additional file 2). This written questionnaire was administered to students in their respective colleges during the college hours.

This questionnaire was first administered to college II and then extended to the other four colleges. Students were allotted 45 min for answering 9 questions (this paper is concerned with only 3 of these questions, i.e., those on the growth and control of microorganisms) and 15 min for the feedback on the questionnaire. The feedback on the questionnaire was checked for internal consistency. Those found to be internally inconsistent were not considered for the analysis, for example, a student reporting that the language of the question was clear and precise and also marking that the language was confusing for the same question. The second questionnaire, to collect data on students’ career aspirations, was administered online to all 348 students; a total of 192 responses were received.

The third group consisted of 7 in-service teachers, who had been teaching these courses to undergraduate students for at least 10 years. A feedback on the expected challenge level and language of the questions was obtained from them online.

The fourth group consisted of 55 in-service teachers (two of them were also part of the third group) having a range of experience (1–34 years) of teaching undergraduate students; some of them had taught the student participants of our study. These 55 teachers were administered an online questionnaire seeking their perspectives on the current laboratory curriculum as well as the practices followed by them in negotiating the curriculum. Students and teachers volunteered their participation in the study, and we did not incentivize or compensate them in any way.

### Development of the questionnaire

The questionnaire developed for the study consisted of both open-ended and multiple-choice questions on three topics—the eukaryotic cell membrane, evolution, and growth and control of microorganisms. The questions were designed from material common to both Microbiology and Biotechnology courses and covered in their syllabus up to the first year of B.Sc. The questions for each topic were designed to cover more than one concept and test higher order thinking skills. None of the questions asked for mere recall; students were required to justify their answers even in the multiple-choice questions.

The questions were then assigned an expected challenge level for students—easy, moderate, or difficult. This was guided by our expectations based on how often students had encountered and/or were required to apply the concepts during their coursework. Their familiarity with the format of the question was also taken into account (Table 2). Finally, a scoring scheme was prepared to ease the process of grading the responses. The questions were selected, refined, and assigned an expected challenge level independently by the expert. A key input by the expert was whether the questions were indeed testing the concepts that they were designed to assess. The scoring scheme and the coding scheme were also discussed with the expert. The expert later became a part of the research team.

**Table 2:** Questions and the expected challenge level assigned by the researchers

Question	Expected challenge level with reason
A researcher needs to sterilize the following items: (a) a solution of vitamins, (b) contaminated hospital linen, and (c) Petri dishes. Which method(s) will you suggest for each? Give reasons for the method(s) you have chosen.	Easy - Students come across these materials very often in their laboratory sessions and, therefore, also with the methods to sterilize each one of them. It is an extremely basic knowledge that is expected of a microbiology/biotechnology student.
A food preparer did not wash his hands and probably introduced 50 *Escherichia coli* cells in the dish. *E.coli* doubles every 20 min, and the dish has been kept outside for about 4 h. If 200 cells degrade 1 g of the dish, determine how many grams of the dish will be degraded at the end of 4 h.	Moderate - In this question, the students needed to recognize the exponential growth pattern of the introduced bacteria to correctly calculate the total number of cells at the end of the given time period. The question required the application of simple arithmetic logic given the doubling time of the bacteria. The second part of the question that asks for the amount of degraded dish was introduced to avoid asking the final cell count directly. The calculation for the degradation of the dish was not considered for the final scoring: Information that should have been provided for students to calculate the degraded amount was missing in the question (and would have made for a very challenging mathematical exercise).
Give your comments on the following: A doctor has just finished removing a patient’s infected kidney and is hurriedly moving to treat another patient who has suffered from burns. So, he quickly wipes all his instruments with a cotton swab dipped in phenol and proceeds to wash them with soap water, then treats the patient who has burns.	Difficult - This was an open-ended question; very different from the format that students are exposed to in their regular assessments. Also, no specific instruction was provided to students as to what was expected in their comment.

### Data analysis and coding

The researchers independently analyzed and scored responses received from the students of college II to the questionnaire. Minor discrepancies (< 0.5%) were resolved through discussions among the researchers and the expert. The final category and score for each question were arrived at by consensus. The questionnaire was then administered to the other four colleges. It was found that the categories of responses that emerged during analysis of responses from college II were exhaustive: Responses from all colleges could be easily be assigned to these categories. The scoring and coding scheme for the three questions is presented in Table 3.

**Table 3:** Coding and scoring scheme used in the study

Coding scheme			Scoring scheme
Question	Category	Sub-category	Score awarded
Suggesting a method for sterilizing: (a) A solution of vitamins (b) Contaminated hospital linen (c) Petri dishes and giving a reason for the method used (maximum score = 3)	Correct (C)	(a) Correctly identified filtration as the suitable sterilization technique citing the property of vitamins to get denatured upon exposure to heat as the reason for choosing this method.	1
		(b) Correctly identified autoclaving as the suitable sterilization technique and considered the bulk of the material to be sterilized.	1
		(c) Correctly identified autoclaving or hot air oven as the suitable sterilization technique citing that high temperature would lead to denaturation of the proteins of microorganisms resulting in their death.	1
	Partially correct (PC)	Either one of the correct method or a relevant consideration in choosing a method identified	0.5 each for (a), (b), and (c)
	Incorrect (IC)	Incorrect method	0
		Incorrect method, reason correct	0
		Incorrect reason, method correct	0
	Unrelated	–	0
	Not attempted (NA)	–	0
Mathematical problem (maximum score = 2)	Correct (C)	Identified exponential growth of the organism and calculated the number of cells correctly	2
	Partially correct (PC)	Exponential growth of *E.coli* identified correctly but arithmetical errors made	0.5
	Incorrect (IC)	Microbial growth considered to be linear	0
	Unrelated (U)	Random calculation with use of unjustifiable and unexplained numbers	0
	Not attempted (NA)	–	0
Doctor’s procedure (maximum score = 3)	Correct (C)	Burns patient is at a higher risk of infection + technique not sufficient + method of sterilization suggested	1 + 1 + 1
	Partially correct (PC)	Any 1 of the above points mentioned	1
		Burn patient is at a high risk of infection + technique not sufficient	1 + 1
		Burn patient is at a high risk of infection + sterilization needs to be done	1 + 1
		Technique used by doctor is not sufficient + sterilization needed	1 + 1
	Incorrect (IC)	Doctor followed the correct procedure	0
	Unrelated (U)	Random answers unrelated to the question	0
	Not attempted (NA)	–	0

A correlation analysis was performed with the students’ scores in our questionnaire and marks scored in the first year college exams using LibreOffice Calc (available in the LibreOffice software package of a Linux system). The first year college marks used for this study were summative of class assignments, internal college exams, and final university exams for MB/BT courses as has been stated in their first year transcripts. Further, we examined the nature of questions appearing in their routine assessments by categorizing them based on revised Bloom’s taxonomy, as described by Krathwohl (2002). A total of 43 questions (32 theory + 11 laboratory) which dealt with the topic of growth and control of microorganisms were identified from five question papers spanning 2 years (2015–2017). These questions along with the three questions on growth and control of microbes from our questionnaire were independently categorized by three members of the research team. To validate our categorization, these 43 questions along with our three questions, included in random order, were shared with two experts having several years of experience in teaching and research and some familiarity with the undergraduate course curriculum. The experts categorized them individually without being aware of the source of the questions. Inter-rater reliability (IRR) was calculated in terms of Krippendorff’s alpha (K-alpha) using a software described by Krippendorff and Craggs (2016) and available freely on the website of Krippendorff, K. (Software to compute the reliability of multi-valued nominal data). K-alpha was calculated both before and after merging the two adjacent categories in Bloom’s taxonomy as described in the following section.

## Results and discussion

### Lacunae in students’ understanding of growth and control of microorganisms

**Fig. 1:**
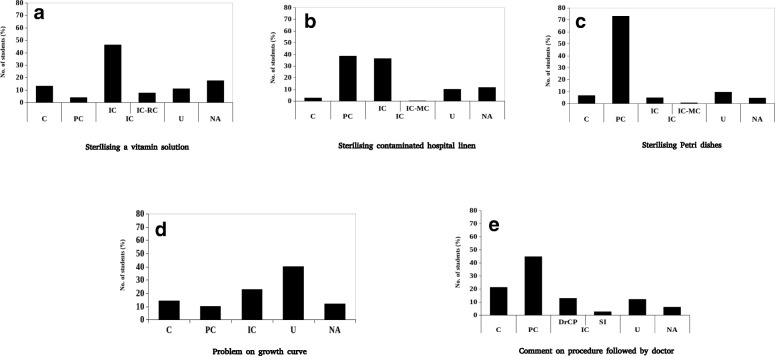
Percentage of student responses in each category for the three questions identified by their keywords: **a**–**c** Subparts of the “easy” question, **d** “moderate” question, **e** “difficult” question. The x-axis represents categories of student responses. *C* Correct; *PC* Partially Correct; *IC* Incorrect; *IC-RC* Incorrect method, correct reason; *IC-MC* Incorrect reason, correct method; *DrCP* Doctor followed the correct procedure; *SI* Soap is an irritant; *INC* Incomplete; U Unrelated; *NA* Not Attempted

The range of student responses to just three questions on growth and control of microorganisms brought out several lacunae in their understanding of the topic. The percentage of responses of second year students in each category for the three questions is presented in Fig. 1, and a summary of their conceptions is presented below.

#### Disinfection vs. sterilization

A major lacuna that became evident in students’ understanding was that they were unable to differentiate between two very distinct processes—sterilization and disinfection. Student responses to two parts in the “easy” question, and the “difficult” question indicates that these two processes were understood to be identical by them. For example, in the former, students responded that contaminated hospital linen could be sterilized using detergents or a disinfectant solution. Curiously, some students also suggested the use of refrigeration as a technique for sterilizing vitamins, perhaps confusing it with preservation. In case of Petri dishes, a variety of suggestions were obtained—using disinfectants such as sodium hypochlorite, phenols, alcohol, washing with detergent, boiling, keeping in an incubator, or increasing the pH. In the latter question, students were explicitly asked to comment on the situation (i.e., the procedure followed by the doctor). The incorrect responses were those that equated disinfection (the procedure followed by the doctor) to sterilization (the procedure that should have been followed by him). A smaller fraction (3%) in this category, in fact, commented only on the use of soap—that it should not have been used as it is an “irritant.”

#### Material to be sterilized not considered

In suggesting a method of sterilization for contaminated hospital linen and a vitamin solution, students did not take into account whether the method would be suitable for sterilizing the given material. For example, students proposed the use of UV radiation or laminar flow, clearly not considering the bulk of the material or that the entire surface area of the linen may not get exposed to UV light. In case of vitamin solution, 46.3% of respondents failed to acknowledge the heat labile nature of vitamins and suggested the use of a hot air oven or an autoclave. It was intriguing to note that a smaller fraction (7.8%) of students who did recognize the heat labile nature of vitamins still proposed incorrect methods. Some advocated against the use of a hot air oven because of its high temperatures (between 160 and 200°C). Instead, they recommended that the solution be autoclaved or heated at lower temperatures (between 50 and 60 °C) in order to prevent degradation of vitamins, failing to take into account that high temperature (121.6°C) exposure during autoclaving will also lead to degradation of vitamins or that sterilization will not occur at lower temperatures.

#### Familiarity with the method but not the principle

A large fraction of students’ responses to the subparts of the “easy” question was partially correct (the method was written without a justification). Such results suggest that students appear to be familiar with many methods used for sterilization but are only able to recall the names without actually understanding the principle of the technique.

#### Methods suggested linked to frequency of use

Students responded to the “easy” question by suggesting, irrespective of the material to be sterilized, some commonly used methods such as autoclaving, using a hot air oven, or a laminar airflow hood—equipment that has a conspicuous presence in an undergraduate laboratory. They seem to be less sure about methods such as membrane filtration, which, although taught in theory classes, is not a commonly used technique for sterilization in their laboratories.

#### Problems with solving a mathematical question

The objective of designing this question was to gauge whether students are able to acknowledge the exponential growth of the microbial population and are able to calculate the total cell count after a given period of time. Only 14.5% of the students were able to calculate the final cell count correctly and another 10% (partially correct (PC)) were at least able to recognize the exponential growth pattern. It was noted that almost a quarter of the students considered the microbial growth pattern to be linear while almost half of the students did random calculations unrelated to the question. This is particularly concerning because students were expected to be familiar with microbial growth kinetics which is dealt with in their curriculum in detail, both during the laboratory (five 3 h sessions), and theory (15 lectures for 50 min each) classes (University of Mumbai n.d). Disturbingly, students had difficulties with basic mathematical operations like multiplication and division.

#### Using inappropriate terminology

Students who wrote a correct response for sterilization of vitamin solution suggested filtration as the method for sterilizing vitamins citing heat lability of the solution as the reason. However, the explanation most of these students gave was that heat cannot be used because it would “denature” vitamins, as opposed to “degrade”. Here, “denaturation” would be an incorrect terminology as it is used in the context of loss of tertiary structure of proteins when exposed to heat or some chemicals, leading to loss of function; the primary structure, however, is maintained. Degradation, on the other hand, is the *breaking down* of the primary structure, leading to an irreversible loss of both structure and function. It is quite possible that the students associated the term “heat labile” to “denaturation” of proteins, thus using the same term while describing another heat labile substance—vitamins. It would be interesting to investigate if the students assumed a structural similarity between the two biomolecules. We would like to highlight that marks were not deducted for the inaccurate use of the term “denaturation” instead of “degradation” to describe the degradation of vitamins because anecdotal evidence suggests that during teaching, vitamins and proteins are often mentioned together as examples of heat labile substances. This may have led to the incorrect use of terminology.

### Students’ and teachers’ rating of the difficulty of questions

Rating of the difficulty of questions was sought from all participating students (group 1 + group 2) and 7 teachers (group 3). We refer to the students’ responses as “rating of the difficulty” while the rating by teachers and researchers is referred to as the “expected challenge level” for reasons that are self-explanatory. Second year students’ rating of the difficulty is shown in Table 4.

**Table 4:** Students’ (group 1—second year) rating of the difficulty of our three questions (% of responses)

	Question		
	Suggest sterilization technique	Problem on growth curve	Comment on procedure followed by the doctor
Number of valid responses received	274	255	262
Difficulty level			
Very easy	5.84	5.10	5.34
Easy	43.43	26.27	45.04
Neither easy nor difficult	42.70	41.18	41.98
Difficult	7.66	23.14	7.25
Very difficult	0.36	4.31	0.38

For the question marked “easy” by the researchers, a majority of students, as well as the teachers, have marked this question as easy or moderately difficult; more or less in alignment with the challenge level assigned by the researchers. One reason why this question could have been considered to be of moderate difficulty by some students and 5 out of 7 teachers is that the question did not just ask for a mere recall of the technique but also demanded an explanation for suggesting that technique. This would require a thorough understanding of the principles behind the techniques.

Unlike the researchers, all the 7 teachers considered the problem on the growth curve to be “difficult” from the point of view of the second year students, stating that “students find any question where some application of knowledge is required, difficult.” This finds corroboration in the student responses, where a large percentage (23% + 40.2%) of them answered this question incorrectly owing to errors in basic mathematical calculations and gaps in the understanding of the growth curve. This indicates that the teachers did identify that students face difficulties in the application of basic quantitative skills to biological concepts and hence categorized the question as “difficult”. Intriguingly, students (*n* = 255), on the other hand, gave a mixed response to this question (easy—31.37%, moderate—41.18%, difficult—27.45%).

While the researchers marked the question asking students to comment on the procedure followed by the doctor as difficult due to its open-ended nature, students have marked it to be of easy/moderate difficulty. Only a very small fraction (about 8%) marked this question to be difficult/very difficult while the rest found it easy or moderate. This is generally consistent with their responses to the question where 66% of students gave either a correct (21%) or partially correct (45%) response. Five of the teachers marked the question to be moderate/difficult and reported that students would find it difficult to answer questions which required the application of concepts and suggested that we “pose a proper question, like “do you think this is the correct practice? Justify.” Students will not know what is expected of them.”

The slight difference between the teachers’ and researchers’ categorization of questions could be attributed to, perhaps, the difference in perspectives. The teachers have marked the category based on their knowledge about what students do know whereas the researchers have categorized the questions based on what students in their second or third year would be *expected* to know after having studied these topics in their classes.

### Curricular issues

An analysis of students’ performance in our test led us to explore the potential reasons for lacunae in their conceptual understanding of growth and control of microorganisms.

#### Poor correlation between performance in the college exams and on our questionnaire

**Fig. 2:**
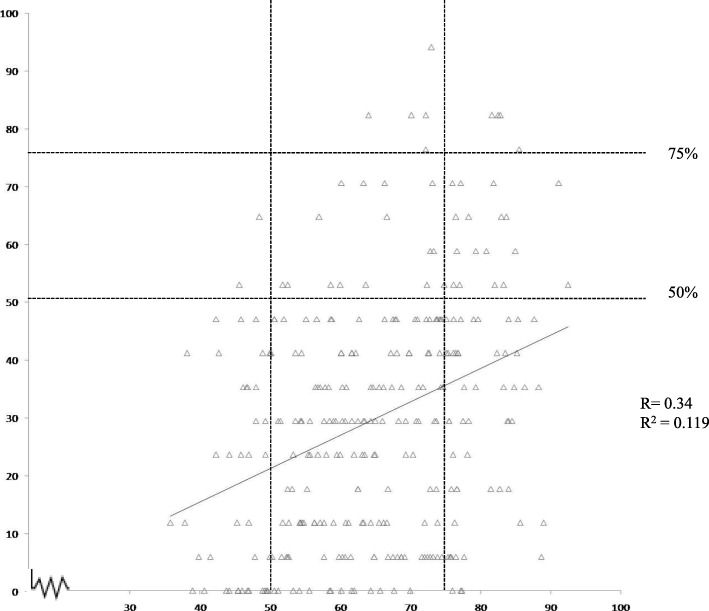
Correlation between scores obtained in university exams and three questions on growth and control of microorganisms from our questionnaire. The *x*-axis represents the percentage of marks obtained by students of group 2 in their first year of the degree course (MB/BT). The *y*-axis represents the percentage of marks obtained by students in three questions on the growth and control of microorganisms from our questionnaire

A correlation analysis was performed between group 1 students’ marks on questions on growth and control of microorganisms in our questionnaire and their scores in the first year University exams in Microbiology (MB) and Biotechnology (BT) courses. The correlation coefficient (*R*) was found to be 0.34 (determination coefficient (*R*^2^) of 0.119), indicating a weak correlation between their scholastic achievement and the marks obtained on our questions as depicted in Fig. 2 (The boundaries of intervals of below 50% and above 75% have been denoted by dotted lines for ease of comparison). As per University norms, the minimum passing marks are 33%, and a score above 75% is considered passing with distinction. It was noted that only four students with college scores above 75% could score similarly (above 75%) in our test while most of the other students with high college marks scored below 50% in our test. However, most students who scored poorly in college exams did poorly on our questions as well, and about 10% of the students scored similarly on both.

Further, we examined some question papers from routine assessments of undergraduate students during the first year of the degree exam and categorized them based on cognitive levels of revised Bloom’s taxonomy. Out of the total of 46 questions (43 from university exams + 3 from our questionnaire), as many as 14 questions were clearly categorized under “remember” (R) and 6 under “understand’” (U) by all evaluators (Additional file 3: Table S1). “R/U” was assigned to 14 other questions, all from university exams: Although some of these questions appeared to require an understanding of the concept, they could well be answered by mere replication of information easily available in most reference books, leading to some ambiguity in assigning the category. This indicates that most of the assessments that students are regularly exposed to are designed either to test mere recall of facts or understanding, but cannot distinguish between the two. Hence, the categories R and U were merged for calculation of K-alpha for IRR. The K-alpha was calculated to be 0.692 without merging the categories R and U which increased to 0.882 after merging the categories. In both cases, K-alpha was found to be above 0.6 showing a good agreement between the raters. Only six questions, notably all from practical exams, could be placed in “apply” (AP) or “analyze” (AN) category. The researchers and expert 1 did not identify a single question that required critical thinking and could be placed in higher order thinking category while expert 2 assigned three questions (nos. 11, 20, and 30) to either “evaluate” (E) or “create” (CR) categories. These questions asked students to justify or write a short note on a particular topic which the researchers and expert 1 categorized as either “remember” or “understand”. Phadnis and Pandit (2011) had pointed out that conventional assessment systems in Mumbai University fail to test conceptual understanding or critical thinking skills. Our results are in agreement with their findings. The questions developed by us, however, ranged from “understand” to “evaluate”, i.e., there was a gradual increase in the thinking order (Additional file 3: Table S1). Overall, the students seem to have performed better in a predictable, rote-learning-based assessment while faring poorly in our test, which had questions that tested conceptual clarity and critical thinking skills.

#### Revisiting the concepts: did it make a difference?

**Fig. 3:**
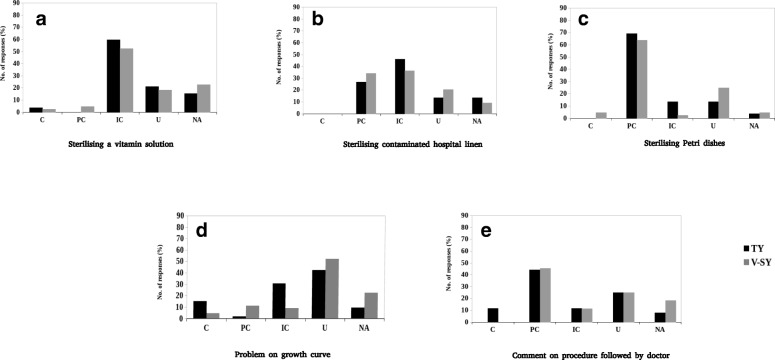
Percentage of student responses in each category for the three questions identified by their keywords: **a**–**c** are subparts of the “easy” question. **d** "moderate" question, **e** "difficult" question. The *x*-axis represents categories of student responses. *C* Correct; *PC* Partially Correct; *IC* Incorrect; *U* Unrelated; *NA* Not Attempted; *TY* Third-year students from college V; *V-SY* Second-year students from college V

As already mentioned in the section on “Participants in the study and data collection”, the same questionnaire was administered to 52 third year students of college V. The curriculum of the final year of the undergraduate course has advanced topics such as industrial and food microbiology (both in theory and laboratory exercises) where they have an opportunity to revisit their ideas of sterilization. They also have a course on microbial growth kinetics where students study various aspects of “microbial growth” and ways to measure it. It is important to note that one section in this course deals specifically with the mathematical expression of microbial growth (see Additional file 4: Table S2). Even though the third year students were expected to have a better understanding of the concepts, the distribution of their responses in various categories was, however, found to be similar to those of the second year students of the same college (Fig. 3). On the question asking for sterilization methods for three different items, students (n = 44), largely, responded that it was easy/very easy (47.73%) or moderate (45.45%). Even though very few students found this question to be difficult, not a single student could answer correctly for the subparts on Petri dish and hospital linen while only two students were able to write the correct method and reason for sterilizing a vitamin solution. Again, mixed responses were obtained (n = 46) for the problem on the growth curve—41.30% thought it was easy/very easy, 32.61% marked it to be moderately difficult, whereas the remaining thought it was difficult/very difficult. As shown in Fig. 3, a larger fraction (15.40%) of third year students, as compared to 5% of second year students, correctly computed the number of cells; however, 30.80% considered a linear growth curve. Another 42.30% made random calculations completely unrelated to the problem. Lastly, in the question asking for their comments on the procedure followed by the doctor (n = 45), only 11.11% of students reported that the question was difficult/very difficult while 46.67% reported it to be easy/very easy. Surprisingly, only 11.5% of students were able to give a completely correct response to this question.

Even though the third year students’ responses were small in number and were collected only from one college, the absence of a single correct answer for the first question and a large number of students failing to acknowledge the exponential growth pattern of microorganisms despite taking additional courses is noteworthy and not expected of this group.

#### Teaching practices and teachers’ perspective on laboratory curriculum/student learning

Table 5 shows the responses of group 4 teachers (*n* = 55) to that part of the questionnaire that required marking on a Likert scale. Open-ended questions from the questionnaire are being discussed in the text. An analysis of teachers’ responses to our questionnaire probing teaching practices revealed that students have little involvement in the design of or in the preparation of laboratory material for experiments. For instance, as part of their experiments, students are routinely required to grow microorganisms in the laboratory which require an application of sterilization methods. In response to the question asking teachers to list the role(s) and contribution(s) of the laboratory staff, students, and teachers in planning and preparation of the practical sessions, all teachers reported that preparation and sterilization of material needed for the experiments are done by laboratory staff; the role of the students was listed largely as “performing the experiment”.

**Table 5:** Teachers’ (group 4, *n* = 55) responses to the questionnaire on their practices and the effectiveness of the laboratory curriculum

	Statements	No. of teacher responses (%)				
Section 1: Indicate how often the following happens						
		Always	Very often	Sometimes	Rarely	Never
1	Students are given the protocol	60.00	32.73	7.27	0.00	0.00
2	Students are encouraged to read research articles related to the experiment	41.82	27.27	25.45	5.45	0.00
3	Students bring protocols from research articles/books	1.82	16.36	41.82	30.91	9.09
4	Teachers have 2–3 different protocols, from students, for the same experiment	5.45	7.27	32.73	34.55	20.00
Section 2: Indicate your level of agreement with the following statements						
		Strongly agree	Agree	Neither agree nor disagree	Disagree	Strongly disagree
1	Students only use the protocols printed in the journal*	25.25	40.00	18.18	16.36	0.00
2	Students do not understand research articles	9.09	25.45	18.18	40.00	7.27
3	Practicals are conducted in alignment with what students are learning in theory	21.82	47.27	18.18	10.91	1.82
Section 3: To what extent does the current laboratory course have a positive impact on						
		High	Medium	Unsure	Low	No
1	Learning technical skills	38.18	58.18	3.64	0.00	0.00
2	Student engagement with underlying principles	34.55	65.45	0.00	0.00	0.00
3	Understanding research methodology	10.91	34.55	32.73	21.82	0.00
4	Building a link between theory and practicals	21.82	60.00	9.09	9.09	0.00
5	Attracting them to research	14.55	43.64	25.45	12.73	3.64
6	Acquiring quantitative skills	12.73	45.45	34.55	5.45	1.82
7	Developing analytical skills	12.73	43.64	25.45	18.18	0.00
8	Ability to deal with unexpected situations	9.09	38.18	30.91	21.82	0.00
9	Motivation to pursue science	20.00	60.00	14.55	3.64	1.82

*Handbook used in the laboratory to record observations and write conclusions; contains the aim, principle involved, and the detailed method to be followed

An overwhelming majority (about 80%) of teachers have also reported that instances of students consulting research articles or books for suggesting experiment protocols were rare. This was particularly surprising because a large fraction (69%) of teachers reported that they always/very often encourage students to read research articles related to the experiment. Also, when asked to opine on the statement that “students do not understand research articles”, a substantial fraction (47.3%) disagreed with the statement. The reason for students not referring to protocols presented in research articles can be inferred from teachers’ responses where a large section (82.7%) has reported that students are always, or at least very often, given the protocol to be followed in a particular laboratory session. A substantial fraction (65.2%) of teachers also agreed that these protocols are the ones printed in their laboratory journal (handbook). Laboratory journals generally contain protocols that have been standardized to obtain a particular result and do not allow for any variation. Based on these findings, we surmise that teachers encourage students to read articles as secondary sources of information and not for developing a hypothesis or for designing experiments.

The questionnaire also consisted of a section probing the level of positive impact of the laboratory curriculum on some aspects of student learning. The teachers reported that the laboratory curriculum has a high/moderately positive impact in building a link between theory and laboratory (81.8%). Consistent with this response, teachers (69%) agreed that they conduct laboratory sessions in alignment with what students are studying in the theory classes. They also reported a high/medium positive impact on the learning of technical skills (96.4%), allowing for student engagement with the principles of the methods they are employing in the experiment (100%), attracting students to research (58.2%), providing with opportunities to develop quantitative skills (58%) and analytical skills (56.4%), and motivating students to pursue science for further education or careers (80%). However, the level of positive impact was, largely, reported by them as moderate or that they were unsure of the impact in aspects such as providing an understanding of research methodology (67.3%) and making students competent to deal with unexpected situations (69.1%).

In summary, the teachers are of the view that the laboratory curriculum and their practices satisfactorily aid in student learning. However, this view is not supported by the findings of our study.

### Alignment between students’ aspirations and teachers’ views of their preparedness

**Fig. 4:**
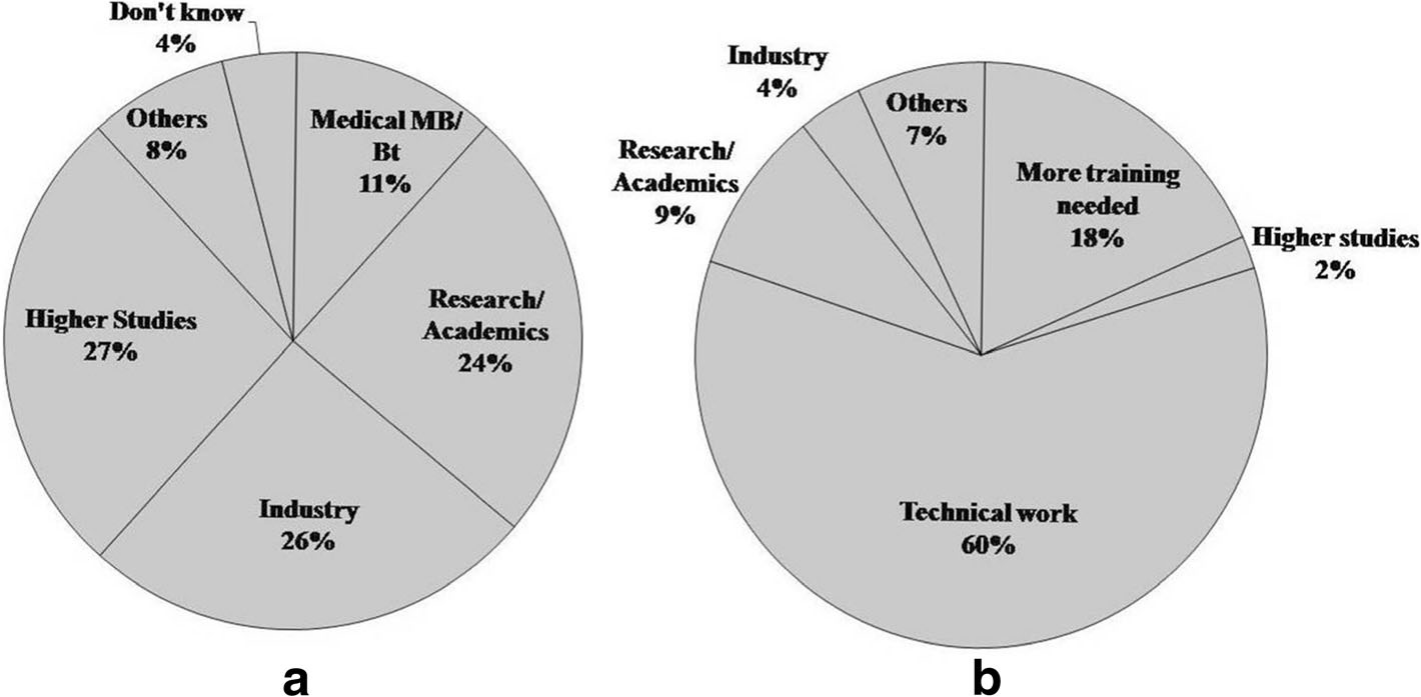
**a** Students’ (*n* = 192) reported career choices. **b** Teachers’ (*n* = 55) report on career options suitable for students after the degree course

Among the 192 students who responded to the questionnaire on career aspirations, the most favored career options were research/academics (24%) or employment in an industry (26%) (food industry being a favorite) (Fig. 4a). A smaller fraction (11%) reported that they wanted to work in the medical microbiology/biotechnology sector. For success in any of these fields, a clear understanding of basic concepts such as growth and sterilization of microorganisms is crucial. Our findings suggest that their lack of conceptual clarity may act as an impediment in carrying out basic duties effectively as part of their jobs. We also asked teachers what their students could do in a research laboratory, without any further training after completion of the 3-year degree course (Fig. 4b). A sizeable fraction (~ 60%) opined that students would be able to work as technicians/laboratory assistants—preparing solutions and reagents, handling equipment, or performing some experiments done by them in their laboratory sessions. Another section (18%) reported that students would not be able to do much after their undergraduate degree and would need some more training before they can work in a research laboratory. A very small percentage (9%) of teachers thought that students could work in the field of academics/research/industries.

## Conclusions

The analysis of students’ conceptions of growth and control of microorganisms revealed several gaps in their understanding of this basic topic. A similar performance by third year students is further indicative of a systemic failure—in both teaching and assessment—to build even the basic practicable knowledge expected of an undergraduate student enrolled for a degree in microbiology or biotechnology. The results corroborate the finding of a previous study by Phadnis and Pandit (2011) that the conventional assessment system in Mumbai University has led to a practice of superficial learning suited only for passing the university examinations which are typically designed to test lower level cognitive skills. Our study highlights a disconnect between theory and practice in the curriculum which, along with the shortcomings of the assessment system, can explain these gaps in students’ understanding. Teachers in our study reported that the laboratory curriculum is aligned with the theory, and it helps students to understand the underlying principles. However, students’ familiarity with a variety of methods for sterilization but their inability to cite reasons for their choice, even for something as commonly used in the laboratory as a Petri dish, is indicative of a disconnect between the two. Further, even though the laboratory examination questions were categorized as “apply”/“analyze”, the laboratory routine itself does not foster science process skills necessary for a career in research or industry. As noted in the section on teachers’ practices in the laboratory, there is very little student involvement in the design, planning, and preparation of the experiments for laboratory sessions, which is rather unfortunate. The laboratory classes are typically structured to follow “cookbook” protocols which do not allow for hypothesis testing or problem-solving but merely serve as validation routines for some concepts covered in the theory classes. The theory and laboratory sessions are also scored separately in the university exams creating a further disconnect. Such a segregation of theory classes from laboratory sessions often leads to students’ lack of conceptual clarity (Abrahams and Millar 2008). An integrated curriculum which fosters experiential learning and encourages learning by research (Kolb 1984; Healey and Roberts 2004; National Research Council (NRC) 2010) is probably what is required to revamp undergraduate biology education in India (such issues have been highlighted in the discourse on necessary nationwide reforms in higher education). It is interesting to note that even though the majority of teachers believed that the laboratory curriculum is effective in attracting students to research or related careers in science, only a small percentage of teachers reported research/academics as a suitable career option for students after the degree course. This is in sharp contrast to students' own reports in which research and industry have been cited as the preferred career options.

Another area of concern is the rather dismal performance in the question requiring quantitative reasoning; indeed, biology students’ problems with mathematics are well documented (Speth et al. 2010; Feser et al. 2013; Hester et al. 2014). The teachers reported that the current laboratory curriculum has a positive impact on building analytical and quantitative skills, while they rated the question on the growth curve as difficult. They also reported that students are used to answering direct questions and find it difficult to deal with application-based questions, which was reflected in the performance of students. If analytical and quantitative skills were being fostered in the laboratory course, this problem should not have been so challenging on the questionnaire. There has been increasing evidence that teaching mathematics to biology majors in the context of biological problems enhances their mathematical skills (Bialek and Botstein 2004; Herreid 2014). Simple exercises in the biology laboratory could be used to generate data which can be examined and discussed in theory classes. A practicable approach would be to intercalate theoretical concepts with laboratory-based activities and thus inculcate problem-solving skills (Kloser et al. 2011). For instance, a hands-on experience in culturing microorganisms where sterilization of the laboratory equipment and growth media is an essential basic step would help build a better understanding of the concept. It is not too difficult to design problems where students actually test several methods of sterilization and figure out the suitability of a particular method for different materials in the laboratory. Similarly, the effectiveness of different disinfectants may be tested by coupling the activity with plotting the growth curve and calculating for the residual bacterial load. An alternative approach could be to consolidate the undergraduate laboratory curriculum across the 3 years into small research projects that can be carried out by students in groups. Evidence suggests that project-based learning through hands-on experience can foster the development of problem-solving skills and lead to better learning (Walker et al. 2015; Wei and Woodin 2011).

In summary, teachers’ expectation (as reflected in teachers’ rating of the expected challenge level of our questions) of students’ performance was consistent with our assessment of students’ conceptual understanding. Their evaluation of students’ preparedness for careers in these fields is also consistent with our findings. We note, however, that there was a striking mismatch between students’ stated career aspirations and their preparedness for them. Yet the curriculum, particularly aspects related to laboratory practice, does not seem to address these issues.

Our findings on students’ conceptions, together with teachers’ responses on the laboratory curriculum and evidence for inadequacies in the assessment system, highlight the need for reforms in the teaching-learning process in the undergraduate biology courses for creating a meaningful learning experience for students. This would be the first step in helping students to achieve competence for careers they aspire to pursue. We have also pointed to feasible changes in the curriculum which can aid both curriculum designers and in-service teachers in improving the current practices. We hope we have demonstrated the value of focused discipline-based education research in pointing out specific measures towards reforms in higher education.

## Additional files

Additional file 1: **Figure S1.** Distribution of students’ marks in (a) grade 12 and (b) first-year (MB/BT). (EPS 45 kb)

Additional file 2: Questionnaire to seek students’ rating of the difficulty of content and language of the questions on growth and control of microorganisms (appearing after every question). (DOCX 23 kb)

Additional file 3: **Table S1.** Categorization of questions based on revised Bloom’s taxonomy. (PDF 185 kb)

Additional file 4: **Table S2.** Syllabus for the topic of growth and control of microorganisms over the 3-year degree course. (PDF 39 kb)

## Abbreviations

AN: Analyze
AP: Apply
B.Sc: Bachelor of Science
BT: Biotechnology
CR: Create
E: Evaluate
*E.coli*: *Escherichia coli*
MB: Microbiology
PC: Partially correct
R: Remember
SY: Second year
TY: Third year
U: Understand
