# Correction to: Is the undergraduate microbiology curriculum preparing students for careers in their field?: an assessment of biology majors’ conceptions of growth and control of microorganisms

**DOI:** 10.1186/s40594-018-0145-0

**Published:** 2018-11-21

**Authors:** Aakanksha Purushottam Sawant, Swapnaja Arvind Patil, Jyotsna Vijapurkar, Needa Nasir Bagban, Deepti Bhushan Gupta

**Affiliations:** 1 Homi Bhabha Centre for Science Education (Tata Institute of Fundamental Research), V.N. Purav Marg, Mankhurd, Mumbai, 400088 India; 2 Present Address: Wildlife Conservation Trust, Mafatlal Center, Nariman Point, Mumbai, 400021 India

## Correction to: International Journal of STEM Education (2018) 5:42 10.1186/s40594-018-0138-z

Following publication of the original article (Sawant et al., 2018), a typesetting mistake was reported. The explanation of part labels d and e have been omitted from the caption of Fig. 1. Figure 1 and its complete caption are given in this Correction article. The original article has been updated.

**Fig. 1:**
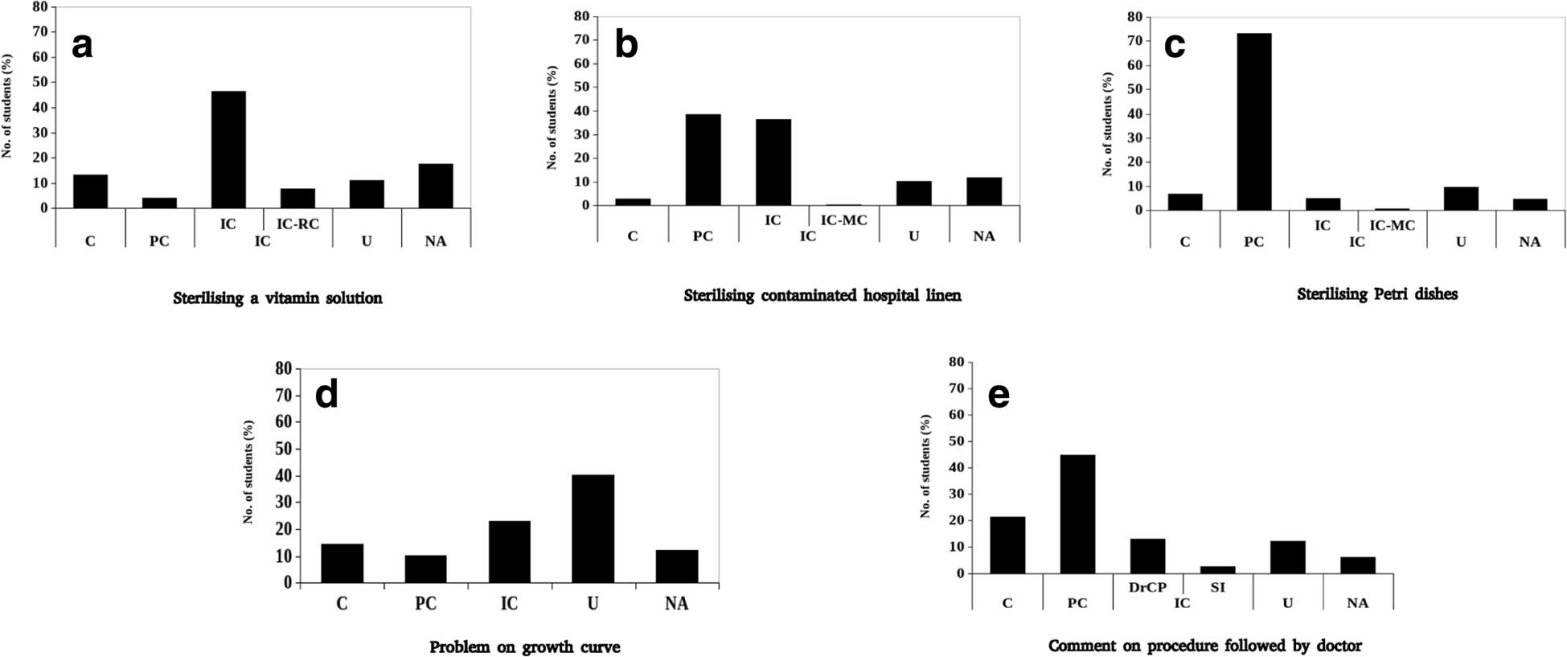
Percentage of student responses in each category for the three questions identified by their keywords: **a**–**c** Subparts of the “easy” question, **d** “moderate” question, **e** “difficult” question. The x-axis represents categories of student responses. C Correct; PC Partially Correct; IC Incorrect; IC-RC Incorrect method, correct reason; IC-MC Incorrect reason, correct method; DrCP Doctor followed the correct procedure; SI Soap is an irritant; INC Incomplete; U Unrelated; NA Not Attempted

The publisher apologises to the authors and readers for the inconvenience.
