# Supplementary material for: Is the undergraduate microbiology curriculum preparing students for careers in their field?: an assessment of biology majors’ conceptions of growth and control of microorganisms
Source: Int J STEM Educ. 2018 Oct 19;5(1):42. doi: 10.1186/s40594-018-0138-z (PMC6310449; doi:10.1186/s40594-018-0138-z)
Supplement: Supplementary file 2 — Questionnaire to seek students’ rating of the difficulty of content and language of the questions on growth and control of microorganisms (appearing after every question). (DOCX 23 kb) [file 40594_2018_138_MOESM2_ESM.docx]

**Additional file 2**

Questionnaire to seek students’ rating of the difficulty of content and language of the questions on growth and control of microorganisms (appearing after every question).

Please tick the appropriate box

| Very easy | Easy | Neither easy nor difficult | Difficult | Very difficult |
| --- | --- | --- | --- | --- |

|  | Yes | No |
| --- | --- | --- |
| Language of the question was clear and precise |  |  |
| Terms used in the question were unfamiliar |  |  |
| Language was confusing |  |  |
