# Supplementary material for: Is the undergraduate microbiology curriculum preparing students for careers in their field?: an assessment of biology majors’ conceptions of growth and control of microorganisms
Source: Int J STEM Educ. 2018 Oct 19;5(1):42. doi: 10.1186/s40594-018-0138-z (PMC6310449; doi:10.1186/s40594-018-0138-z)
Supplement: Supplementary file 3 — Table S1. Categorization of questions based on revised Bloom’s taxonomy. (PDF 185 kb) [file 40594_2018_138_MOESM3_ESM.pdf]

Questions		Researcher 1	Researcher 2	Researcher 3	Expert 1	Expert 2
**Theory**						
1	___________is the size of millipore filters.	R	R	R	R	R
2	Define – Sterilization	R	R	R	R	R
3	The time required to kill 90% of the microorganisms or spores in a sample under specified conditions is _______.	R	R	R	R	R
4	Give two examples of biological indicators to check autoclave efficiency.	R	R	R	R	U
5	Define – Bactericidal.	R	R	R	R	R
6	Explain in brief how aldehydes and halogens control microbial growth	U	U	U	U	U
7	___________is defined as reduction of the microbial population to levels considered safe by public health standards.	R	R	R	R	R
8	Explain how filteration and radiation controls microorganisms.	R/U	U	U	U	U
9	The pore size of a HEPA filter is __________.	R	R	R	R	R
10	Justify that there are many factors which affect activity of antimicrobial agent.	U	U	U	U	E
11	Aqueous solution of formaldehyde is __________.	R	R	R	R	R
12	What are the different physical sterilisation methods?	R	R	R	R	R
13	___________% solution of gluteraldehyde has a wide spectrum of activity.	R	R	R	R	R
14	What should be the properties of an ideal disinfectant?	U	U	U	U	R
15	Explain the construction and working of an autoclave.	R/U	U	U	U	U

16	Give two examples of filters used in sterilisation.	R	R	R	R	U
17	Write short note on use of wet heat to control microorganims.	R	R	R	U	CR
18	Define: Dye.	R	R	R	R	R
19	State whether True or False: Inspissator is used to sterilize media containing egg.	U	R	R	R	R
20	Give the significance of Phenol Co-efficient.	R	R	R	U	R/U
21	Give two examples of Phenolic compounds.	R	R	R	R	U
22	State whether True or False: A bactericide kills all microorganisms.	R/U	R	R	U	R
23	Discuss the construction and working of an autoclave.	R/U	U	U	U	U
24	Write a short note on Ionising radiation and their use in sterilisation.	R/U	R	R	R	CR
25	Explain the mode of action and practical applications of the following chemical antimicrobial agents: Heavy metals and compounds and alcohols.	U	U	U	U	U
26	Give two examples of alcohols used as sterilants.	R	R	R	R	U
27	Give one example for bacteriological filter.	R	R	R	R	U
28	Give two examples of gaseous disinfectant.	R	R	R	R	U
29	Briefly describe how Quarternary ammonium compounds are used as bactericidal agents.	U	U	U	U	U
30	Give the importance of hot air oven.	R	R	R	R	R/U
31	Give two examples of types of radiation.	R	R	R	R	U
32	Give two examples of antiseptic liquids.	R	R	R	R	U

	Laboratory	Researcher 1	Researcher 2	Researcher 3	Expert 1	Expert 2
33	How are dry powders sterilised?	R/U	R	R	R	R
34	Provided to you is a culture suspension. Comment on the growth pattern of this organism in liquid media after 24 Hr incubation?	AN	AN	AN	AN	AN/E
35	Give principle of tyndallization.	R	R	R	R	R
36	Using suitable technique determine the oligodynamic action of copper on the provided pathogenic Gram positive organism.	AP/E	AP	AP	AP	AP
37	Provided to you is a culture suspension. Use a simple lab technique which will preserve the culture using agar medium.	AP	AP	AP	AP	AP/AN
38	Give role of Class III cabinets.	R	R	R	R	R/U
39	A Gram positive skin pathogen has been provided to you. Study the effect of two chemotherapeutic drugs on the culture and comment on your results.	AP/AN	AP/AN	AP/AN	AN	AP/AN
40	Using suitable technique determine the action of heavy metals on the provided pathogenic Gram positive organism.	AP	AP	AP	AP	AP
41	How are bandages sterilised?	R/U	U	U	U	R
42	Provided to you are two dyes. Their role as inhibitory agent against Gram positive organism has to be ascertain. Use a suitable agar plate technique and report your findings	AP	AP	AP	AP	AP/AN/E/ C R
43	Give one example of a lab disinfectant.	R	R	R	R	U

Questions developed by the researchers		Researcher 1	Researcher 2	Researcher 3	Expert 1	Expert 2
44	A researcher needs to sterilize the following items: (a) A solution of vitamins, (b) contaminated hospital linen (c) petri dishes. Which method(s) will you suggest for each? Give reasons for the method(s) you have chosen.	U	U	U	U	U/E
45	Give your comments on the following: A doctor has just finished removing a patient's infected kidney and is hurriedly moving to treat another patient who has suffered from burns. So he quickly wipes all his instruments with a cotton swab dipped in phenol and proceeds to wash them with soap water, then treats the patient who has burns.	E	E	E	E	E
46	A food preparer did not wash his hands and probably introduced 50 *E.coli* cells in the dish. *E.coli* doubles every 20 minutes and the dish has been kept outside for about 4 hours. If 200 cells degrade 1 gram of the dish, determine how many grams of the dish will be degraded at the end of 4 hours.	AP	AP	AP	AP	AP

Key:

R: Remember U: Understand
E: Evaluate CR: Create
AP: Apply AN: Analyse

Supp. Table 1 Categorisation of questions based on revised Bloom’s taxonomy
