# Supplementary material for: Is the undergraduate microbiology curriculum preparing students for careers in their field?: an assessment of biology majors’ conceptions of growth and control of microorganisms
Source: Int J STEM Educ. 2018 Oct 19;5(1):42. doi: 10.1186/s40594-018-0138-z (PMC6310449; doi:10.1186/s40594-018-0138-z)
Supplement: Supplementary file 4 — Table S2. Syllabus for the topic of growth and control of microorganisms over the 3-year degree course. (PDF 39 kb) [file 40594_2018_138_MOESM4_ESM.pdf]

Syllabus (University of Mumbai) for the topic of growth and control of microorganisms over the three-year degree course

Year	Microbiology syllabus	Biotechnology syllabus
First-year	**Control of microorganisms**<br>Definition of frequently used terms & Rate of microbial death,Factors affecting the effectiveness of antimicrobial agents & Properties of an ideal disinfectant<br>Evaluation of disinfectant –Tube dilution & Agar plate techniques, Phenol coefficient etc , Tissue toxicity index<br>Physical methods of microbial control<br>Chemical methods of microbial control - mechanism & advantages &disadvantages (if any)<br>applications.<br>Chemotherapeutic agents List types of agents active against various groups & mention the site of action (Detailed mode of action not to be done)<br><br>**Microbial Growth**<br>Definition of growth, Mathematical Expression, Growth curve<br>Measurement of growth Direct microscopic count – Breed's Petroff – Haussercountingchamber, Hacmocytometer.<br>Viable count – Spread plate and Pour plate technique<br>Measurements of cell consituents.<br>Turbidity measurements – Nephelometer and spectrophotometer techniques<br>Measurements of cell constituents Synchronous growth, Continuous growth (Chemostat and Turbidostat)<br>Growth yield-Influence of environmental factors on growth.<br>Microbial growth in natural environment.<br>Counting viable non-culturable organisms-Quorum sensing techniques	**Sterilization Techniques**<br>Definition : Sterilization and Disinfection.<br>Types and Applications<br>Dry Heat, Steam under pressure, Gases, Radiation and Filteration<br>Chemical Agents and their Mode of Action-Aldehydes, Halogens, Quaternary Ammonium Compounds, Phenol and Phenolic Compounds, Heavy Metals, Alcohol, Dyes, and Detergents<br>Ideal Disinfectant. Examples of Disinfectants and Evaluation of Disinfectant<br><br>**Nutrition, Cultivation and Enumeration of Microorganisms**<br>Growth and Enumeration<br>Growth Phases, Growth Curve.<br>Arithmatic Growth and Growth Yield.<br>Measurement of Growth. Chemostat and Turbidostat<br>Enumeration of Microorganisms - Direct and Indirect Methods<br>Preservation of Cultures - Principle and Methods. Cryogenic Preservation<br>Advantages and Limitations

Second-year	**Air & Fresh Water Microbiology**<br>Air sanitation - methods and application<br>Potable water: Definition, water purification and pathogens transmitted through water.<br>Modern waste water treatment: Primary, Secondary and tertiary treatment.<br><br>**Industrial, food, and dairy microbiology**<br>Principles and methods of primary and secondary screening.<br>Microbial growth in foods:<br>General principles of food preservation (principle of each method and example of foods only): High temperature, low temperature, drying, radiations and food additives and preservatives (tabular representation)<br>Pasteurization of milk-LTLT, HTST method	**Introduction to fermentors**<br>Sterilization: Maintenance of aseptic conditions (Media and fermentor), Inoculum development: Addition of inoculum, nutrients and other supplements.<br><br>**Microbial growth kinetics**<br>Phases of Growth curve. Direct and indirect methods of measuring growth, Mathematical nature and expression of growth, Efficiency of growth, Synchronous growth, Diauxic growth, Effect of environment and nutrient factors, Chemostat and Turbidostat.<br><br>**Microbiology of air and soil**<br>Air Sanitation- Introduction, Suppression of dust, Effect of mists and sprays, Effect of UV light, Room sanitation.<br><br>**Microbiology of water and waste water**<br>Sanitation of water for domestic use, Preventive treatment, Sedimentation, Coagulation and Flocculation.<br>Filtration – Slow sand filter, Rapid sand filter, Diatomite filter, Reverse osmosis. Disinfection of potable water.
Third-year	**Bioprocess technology**<br>Sterilization - Introduction. Media sterilization (Concept of nabla factor), Design of batch sterilization.<br>Methods of batch sterilization,- Design of continuous sterilization, Methods – Heat, Achievement & maintenance of ascetic condition<br><br>**Quality Assurance & Regulatory Practices**<br>Sterilization Control and Sterility Assurance: Bio-burden determinations<br>Environmental monitoring Sterilization Monitors – Physical, Chemical and Biological indicators<br>Sterility Testing	**Industrial Biotechnology**<br>Primary screening, secondary screening, inoculum and strain development<br>Preservation methods, Pasteurisation
